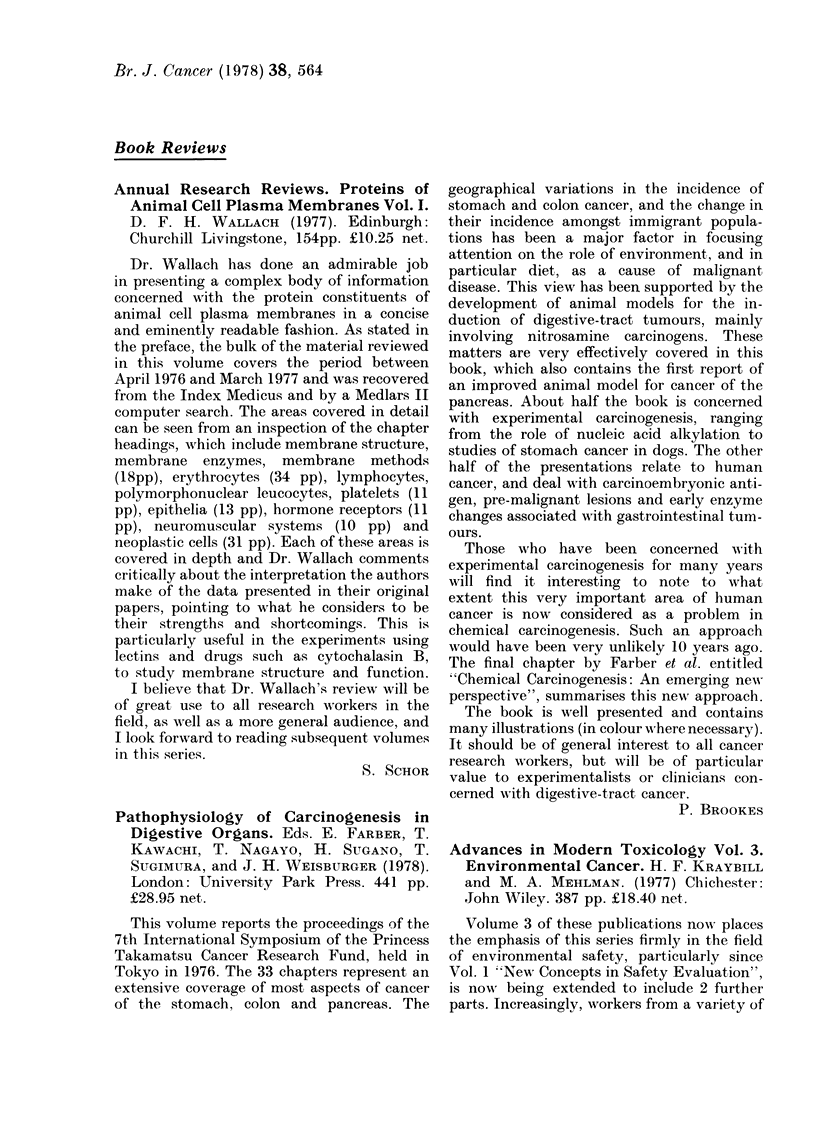# Annual Research Reviews. Proteins of Animal Cell Plasma Membranes Vol. I

**Published:** 1978-10

**Authors:** S. Schor


*Br. J. Cancer* (1978) **38**, 564

### *Book Reviews*

**Annual Research Reviews. Proteins of Animal Cell Plasma Membranes Vol. I.** D. F. H. WALLACH (1977). Edinburgh: Churchill Livingstone, 154pp. £10.25 net.

Dr. Wallach has done an admirable job in presenting a complex body of information concerned with the protein constituents of animal cell plasma membranes in a concise and eminently readable fashion. As stated in the preface, the bulk of the material reviewed in this volume covers the period between April 1976 and March 1977 and was recovered from the Index Medicus and by a Medlars II computer search. The areas covered in detail can be seen from an inspection of the chapter headings, which include membrane structure, membrane enzymes, membrane methods (18pp), erythrocytes (34 pp), lymphocytes, polymorphonuclear leucocytes, platelets (11 pp), epithelia (13 pp), hormone receptors (11 pp), neuromuscular systems (10 pp) and neoplastic cells (31 pp). Each of these areas is covered in depth and Dr. Wallach comments critically about the interpretation the authors make of the data presented in their original papers, pointing to what he considers to be their strengths and shortcomings. This is particularly useful in the experiments using lectins and drugs such as cytochalasin B, to study membrane structure and function.

I believe that Dr. Wallach's review will be of great use to all research workers in the field, as well as a more general audience, and I look forward to reading subsequent volumes in this series.

S. SCHOR